# Cooperation Between the Inflammation and Coagulation Systems Promotes the Survival of Circulating Tumor Cells in Renal Cell Carcinoma Patients

**DOI:** 10.3389/fonc.2019.00504

**Published:** 2019-06-17

**Authors:** Li Wen, Liping Guo, Wen Zhang, Yajian Li, Weixing Jiang, Xuebing Di, JianHui Ma, Lin Feng, Kaitai Zhang, Jianzhong Shou

**Affiliations:** ^1^Department of Urinary Surgery, National Cancer Center, National Clinical Research Center for Cancer, Cancer Hospital, Chinese Academy of Medical Sciences and Peking Union Medical College, Beijing, China; ^2^State Key Laboratory of Molecular Oncology, Department of Etiology and Carcinogenesis, National Cancer Center, National Clinical Research Center for Cancer, Cancer Hospital, Chinese Academy of Medical Sciences and Peking Union Medical College, Beijing, China; ^3^Department of Immunology, National Cancer Center, National Clinical Research Center for Cancer, Cancer Hospital, Chinese Academy of Medical Sciences and Peking Union Medical College, Beijing, China

**Keywords:** renal cell carcinoma, circulating tumor cells, neutrophil extracellular trap, hypercoagulability, C-reactive protein

## Abstract

Most renal cell carcinoma (RCC) patients die from metastasis or recurrence after the spread of cancer to another organ, but the mechanisms underlying the intravascular survival of circulating tumor cells (CTCs) have not been completely deciphered. Additionally, although elevated plasma C-reactive protein (CRP) levels and thrombocytosis are strongly correlated and both indicate a poor prognosis for RCC patients, the bridge connecting inflammation and coagulation remains poorly understood. To explore the complicated relationship among inflammation, the coagulation system and CTC survival, we obtained viable CTC counts and clinical information from 106 treatment-naïve patients. In addition, we performed RNA sequencing on peripheral blood leukocytes from 21 of these patients. Patients with elevated CRP and fibrinogen (FIB) levels had higher CTC counts than patients with normal levels of these indexes. Each pair of the three variables (CTC count, CRP level and FIB level) was positively correlated. According to transcriptomic analysis of blood leukocytes, the functions of the 257 genes identified as being positively correlated with the CTC count indicated neutrophil extracellular trap (NET) formation. Indeed, gene set enrichment analysis (GSEA) suggested that NET formation or increased levels of NET markers would promote CTC viability. Additionally, the calculated NET score was positively correlated with the plasma FIB concentration, and both of these values were increased in patients with elevated CRP levels. Moreover, immunofluorescence staining showed that NETs were entangled with viable renal cancer cells and that the NET frameworks were decorated with NET-derived tissue factor (TF). Finally, analysis of 533 RCC samples from The Cancer Genome Atlas (TCGA) indicated that the NET score and TF value are independent prognostic factors for RCC patients. Collectively, NETs formed by intravascular neutrophils further activate the coagulation system. Both the DNA scaffold sprouted and fibrin net triggered by NETs anchor and shield CTCs from attack. Thus, degrading this framework maybe could destroy the double shelter of CTCs, the pioneers of metastasis.

## Introduction

Although the global incidence of renal cancers ranked 16th among 35 common cancers in 2018 (1), renal cancers carry a poor prognosis. The prognosis of renal cell carcinoma (RCC), the most common kidney tumor, is particularly poor; the 5-year survival rate decreases sharply to 8% in metastatic patients (2). In addition, the recurrence rate is approximately 40% in patients treated with localized disease (3). Therefore, most RCC patients die from the metastasis or recurrence of the cancer after it spreads to another organ. However, little is known about the mechanisms by which circulating tumor cells (CTCs) survive intravascularly, which, in turn, reduces the opportunities to inhibit metastasis.

After shedding from the primary tumor into the circulatory system, a single CTC encounters countless survival challenges, such as attacks by T cells and natural killer (NK) cells, shear forces and oxidative stress (4). However, a few surviving cells can be detected and overtly metastasize, and studies on the mechanisms underlying CTC survival have thus far focused on two mutually dependent aspects. The first aspect involves CTC self-changes, such as the antiphagocytic signal CD47 being overexpressed in circulating colorectal cancer cells (5). The other aspect is the self-arming and self-protection of CTCs with suppressive cellular components; for example, CTCs recruit platelets to aggregate on their surface (6), and neutrophils adhere to circulating liver tumor cells expressing Mac-1 (7).

In RCC patients, elevated plasma C-reactive protein (CRP) levels and thrombocytosis are strongly correlated with poor prognosis and recurrence (8), indicating that inflammation and hypercoagulability may be critical environmental components required for CTC survival in circulation. Regarding inflammation, certain subtypes of neutrophils have been increasingly acknowledged as important components of protumor-associated inflammatory cells (9, 10). Moreover, neutrophils not only mediate inflammation but also initiate the coagulation system by forming a specific structure—neutrophil extracellular traps (NETs) (11).

NETs were first reported as a distinct antimicrobial mechanism in 2004 (12). Specifically, neutrophils are activated with lipopolysaccharide (LPS), phorbol myristate acetate (PMA), interleukin-8 (IL-8), bacteria (13), or granulocyte colony stimulating factor (G-CSF) (14), and histone H3 in neutrophils is citrullinated by the protein arginine deiminase 4 (PAD4) enzyme, which is essential for NET formation. The neutrophil nucleus then undergoes chromatin decondensation and extrudes a meshwork of chromatin fibers. Furthermore, the extruded DNA scaffold is decorated with a variety of enzymes, such as neutrophil elastase (NE), myeloperoxidase (MPO), cathepsin G, matrix metalloproteinase 9 (MMP9) and tissue factor (TF) (13, 15, 16). NET-derived TF, a trigger factor of the extrinsic coagulation pathway, is the link between inflammation and coagulation. Although the initial observation of NET formation was in the context of an innate immune response in an antimicrobial defense process, NETs also participate in various pathologies, such as autoimmune diseases and cancer, and function as a double-edged sword. Most studies suggest that NETs formed in the cancer environment promote tumor progression by enhancing the abilities of cancer cells to invade (14) and proliferate (17), and NETs awaken dormant tumor cells (18). Additionally, NETs formed in the circulatory system can bind CTCs and thus enhance liver cancer metastasis (19, 20), and those formed in the peritoneal cavity can promote colon metastatic colonization (21). Although NETs form and function as tumor promoters in breast liver and colon cancer, their functions in RCC are unclear.

Since RCC is strongly associated with inflammation (22) and frequently accompanied by tumor thrombus, we hypothesize that a bridge links inflammation and thrombosis and that this bridge also shelters CTCs, promoting their survival. The bridge may be the DNA scaffold decorated with neutrophil-derived TF extruded by circulating neutrophils. Thus, we investigated the relationship between the viable CTC counts and peripheral blood leukocyte statuses of RCC patients.

## Materials and Methods

### Collection of Patient Specimens and Clinical Information

In this double-blinded study,106 treatment-naïve patients with renal masses who were going to be treated with surgery at the Cancer Institute and Hospital of the Chinese Academy of Medical Sciences (CAMS) between December 2015 and April 2017 were enrolled. Of the 106 enrolled patients, 95 patients were eventually diagnosed with RCC, 9 patients were diagnosed with benign renal conditions according to the final pathology results, and 2 patients who did not undergo surgery were diagnosed with RCC based on the follow-up results. The RCC stage was designated according to the American Joint Committee on Cancer staging system (23). The clinical and histopathological characteristics of the enrolled patients are shown in Table 1. Additionally, hematological parameters, including neutrophil counts and plasma fibrinogen (FIB) and CRP concentrations, were extracted from laboratory records prior to the operation. The cutoff values for defining “high” and “normal” plasma FIB and CRP levels were determined according to the diagnostic criteria reported by the clinical laboratory at our hospital. 4 ml peripheral blood in K2-EDTA vacuum tubes (BD Biosciences, Franklin Lakes, NJ, USA; cat. no. 367844) for CTC count determination, and RNA were extracted from the remaining peripheral blood for RNA sequencing (RNAseq). This study was approved by the Ethics Committee of the Cancer Institute and Hospital of the CAMS.

**Table 1:** Patient demography.

**Subject category**	***n* (%)**
Total	106 (100)
Benign	9 (8.5)
Renal cell carcinoma (RCC)	97 (91.5)
**Age**	**Median (range)**
Total	55.6 (28–74)
Men	56.1 (31–74)
Women	54.2 (28–70)
**Gender**	
Men	79 (74.5)
Women	27 (25.5)
**Location**	
Right kidney	43 (40.6)
Left kidney	62 (58.5)
Both	1 (0.9)
**Benign subtype**	
Renal cyst	2 (1.8)
Renal oncocytoma	1 (0.9)
Angiomyolipoma	5 (4.7)
Nephrotuberculosis	1 (0.9)
**RCC subtype**	
Clear cell renal cell carcinoma (ccRCC)	84 (79.2)
Papillary renal cell carcinoma (pRCC)	2 (1.8)
Chromophobe renal cell carcinoma (chRCC)	3 (2.8)
Xp11.2 translocation renal cell carcinoma (Xp11_RCC)	3 (2.8)
Others^a^	5 (4.7)
**RCC subtype**	**Stage I/II/III/IV^b^**
all RCC	62/4/17/14
ccRCC	58/3/12/11
pRCC	2/0/0/0
chRCC	2/0/1/0
Xp11_RCC	0/0/3/0
others^a^	0/1/1/3
**Furman's grade**	
I-II	56 (57.7)
III-IV	35 (36.1)
NA	6 (6.2)
**Concentration of preoperative plasma C-reactive protein, mg/dL**	
≦0.6 (normal)	60 (56.6)
>0.6 (high)	23 (21.7)
NA	23 (21.7)
**Concentration of preoperative plasma fibrinogen, g/L**	
≧4 (normal)	64 (60.4)
>4 (high)	16 (15.1)
NA	26 (24.5)

a *Others: two types of pathological subtypes coexisting in one patient or unoperated patients with an undetermined pathological subtype*.

b *Stage I/II/III/IV: number of patients with different RCC subtypes in different clinical stages*.

### Counting and Validation of CTCs With the oHSV1-hTERT-GFP Method

The preoperative CTC counts in 106 patients were measured by laboratory personnel blinded to the diagnosis by the oHSV1-hTERT-GFP method; the detailed protocol for this method is available in publications by Zhang and Wang (24, 25). To verify the feasibility of this CTC detection method in RCC patients, we imaged CTCs using an ImageStream® X Mark II imaging flow cytometer (Merck Millipore, Darmstadt, Germany) with a second marker, a human carbonic anhydrase IX/CA9 APC-conjugated antibody (R&D Systems, Minneapolis, MN, USA; cat. no. FAB2188A) (25).

### Next-Generation Sequencing and Data Processing

Peripheral blood leukocytes from 21 treatment-naïve patients were obtained after platelets were removed by centrifugation, and red blood cells (RBCs) were lysed with erythrocyte lysis buffer (Qiagen, Hilden, Germany; cat. no. 79217). Subsequently, total RNA was extracted from the peripheral blood leukocytes using TRIzol reagent (Invitrogen, Carlsbad, CA, USA; cat. no. 15596018), and the RNA quality was verified by capillary gel electrophoresis on a Bioanalyzer 2,100 instrument using an Agilent RNA 6,000 Nano Kit (Agilent, CA, USA; cat. no. 5067–1511). Next-generation sequencing (RNAseq) libraries were prepared from qualified samples using the NEBNext® Ultra™ RNA Library Prep Kit for Illumina (New England Biolabs, Ipswich, MA, UK; cat. no. E7530L) according to the manufacturer's instructions. Then, eligible libraries were clustered and sequenced on an Illumina HiSeq X 10 platform (150-bp paired-end reads) using HiSeq X Reagent Kits (Illumina, San Diego, CA, USA; cat. no. FC-501-2521).

Adapter sequences and low-quality reads were removed from the fastq files using Cutadapt (26) and Sickle (http://github.com/najoshi/sickle/) software. Clean reads in fastq files were quantified against an Ensembl catalog (GRCh37) at the transcript level using Salmon (27) and aggregated to the gene level using tximport (28); transcripts per million reads (TPM) values were obtained with Salmon gene expression quantification software, and unexpressed genes were removed. The Pearson correlation coefficients between the log2 transformed TPM values and CTC counts were calculated. Genes with a correlation coefficient > 0.4 and a *p*-value < 0.05 were defined as being positively correlated with the CTC count.

Another dataset including the mRNA expression data (RNAseqV2, RSEM) and corresponding clinical information for patients with clear cell renal cell carcinoma (ccRCC) (*n* = 533) was downloaded from The Cancer Genome Atlas (TCGA) (https://tcga-data.nci.nih.gov/tcga/). Subsequent analysis was performed with the log2 transformed mRNA expression values.

### Immune-Related Gene Sets

Gene sets, including gene signatures used for decomposition of the 24 immune cell types, angiogenesis marker genes and antigen presentation signatures, were obtained from a publication by Senbabaoglu et al. (29).Combining known key molecules in the formation of NETs and the genes positively correlated with CTC counts, we defined a gene expression signature for NETs (Table S1) (13, 15, 16, 30–32). In addition, the signature for the coagulation cascade is shown in Table S1. The c7 immunological gene sets were downloaded from the Molecular Signatures Database v6.0 (MSigDB).

### Decomposition of Immune Cell Types (Immunophenoscore)

We used three methods to identify immune cell types and immune phenotypes in peripheral blood. The relative counts of 24 immune cells and their subtypes, the angiogenesis and coagulation activity levels, the antigen presentation conditions, and NET formation were evaluated using the single-sample gene set enrichment analysis (ssGSEA) method and the gene set variation analysis (GSVA) method implemented in the R package “GSVA” (33). The counts and activities of NETs calculated with the ssGSEA method are hereafter referred to as the NET score. The above methods produced scores for each immune cell type and phenotype in every sample, hereafter referred to as the immunophenoscore. In addition, we analyzed the immune phenotypes of the 21 peripheral blood transcriptomes by the GSEA method considering their associated CTC counts as continuous labels with the c7 immunological gene sets (34).

### RCC Cell Line

ACHN cells, which were purchased from the National Infrastructure of Cell Line Resource in China, were cultivated in RPMI-1640 medium (HyClone Laboratories; SH30809.01B) supplemented with 10% fetal bovine serum (FBS; Gibco; 10099–141) and 100 units/mL penicillin and streptomycin.

### Detection of NETs in RCC Patients

Peripheral venous blood (4 mL) from RCC patients was collected into K2-EDTA vacuum tubes (BD Biosciences; 367844). Polymorphonuclear cells (PMNs), mainly neutrophils, were isolated using Polymorphprep (Axis-Shield), and the purity of neutrophils was evaluated as previously described (35). Isolated neutrophils were then resuspended in serum-free RPMI-1640 medium and plated on poly-L-lysine-coated chamber coverslips (Thermo Fisher Scientific; 88–8824) with or without LPS (100 ng/mL) stimulation for 4 h. Next, cells were fixed, permeabilized, blocked and stained with an anti-MPO antibody [2C7] (1:400 dilution, Abcam; ab25989), anti-histone H3 (citrulline R2 + R8 + R17) antibody (1:400 dilution, Abcam; ab5103), anti-TF antibody (1:100 dilution, Abcam; ab48647), Hoechst 33342 (1:1,000 dilution, Invitrogen; H3570), and the appropriate secondary antibody, either goat anti-mouse IgG (Alexa 488) (1:2,000 dilution, Abcam; ab150113) or goat anti-rabbit IgG (Alexa 568) (1:2,000 dilution, Abcam; ab175471).

ACHN cells were stained with PKH26 (Sigma; MIDI26), a general cell membrane-labeling probe that binds lipids, according to the manufacturer's instructions. Subsequently, stained ACHN cells (3,000 cells) were spiked into neutrophils isolated from 4 mL of peripheral venous blood. ACHN cells and neutrophils were cocultured and stained with anti-MPO antibody and Hoechst 33342 according to the abovementioned protocols. Stained chamber coverslips were observed with a confocal laser scanning microscope (Leica TCS SP8).

### Additional Bioinformatic and Statistical Analyses

To distinguish the differentially expressed immune cells between the two groups (stratified by the concentration of plasma CRP, details in Table 1), linear models and empirical Bayes methods were applied with the R package “limma.” Immune cells with an absolute fold change > 1.2 and a *p*-value < 0.05 were defined as differentially active immune cells. Gene Ontology (GO) analysis and hierarchy relation analyses were conducted with the R package “topGO” (36). Gene networks were constructed in Cytoscape 3.6.1 with the STRING plugin (37, 38). The Wilcoxon rank test and Pearson and Spearman correlations were implemented by the R package “stat.” Moreover, survival curves were generated by the Kaplan-Meier method and evaluated with the log-rank test (two-tailed). The hazard ratio was determined by Cox multivariate analysis with the R package “survival.” All tests were two-sided, and p < 0.05 indicated statistical significance.

## Results

### Hematological Parameters Suggested That the Viable CTC Counts Were Related to the Inflammation and Coagulation Statuses of the Patients

**Figure 1:**
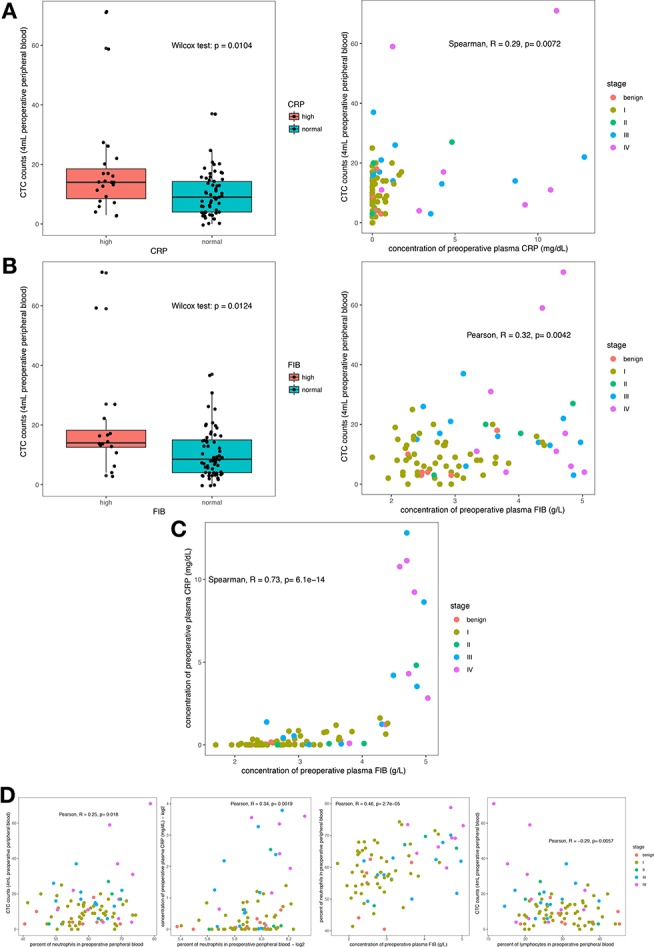
Correlation of hematological parameters (CRP and FIB) with the viable CTC count. CTC counts were increased in the group with elevated circulating CRP levels **(A)** and the group with elevated circulating FIB levels **(B)**, and both had a positive correlation. The plasma CRP and FIB levels showed a strong positive relationship **(C)**. The circulating neutrophil count was positively correlated with the CTC count and with the plasma CRP and FIB levels. However, the circulating lymphocyte count was negatively correlated with the CTC count **(D)**.

We investigated the preoperative viable CTC counts and other clinical characteristics of the 106 enrolled patients (the detailed demographic characteristics of the patients are shown in Table 1) and confirmed that the oHSV1-hTERT-GFP method was feasible in RCC patients (Figure S1). We observed that the CTC counts correlated with the plasma concentrations of CRP and FIB (Figure 1). Specifically, the CTC counts in the group with elevated CRP levels were higher than those in the group with normal CRP levels (Wilcoxon test, *p* = 0.0104), and both variables displayed a positive correlation (Spearman correlation analysis, *R* = 0.29, *p* = 0.0072) (Figure 1A). FIB, a terminal coagulation factor in the coagulation cascade, exhibited a similar pattern; the CTC counts in the group with elevated FIB levels were higher than those in the group with normal FIB levels (Wilcoxon test, *p* = 0.0124), and higher FIB levels were correlated with higher viable CTC counts (Pearson correlation analysis, *R* = 0.32, *p* = 0.0042) (Figure 1B). In addition, CRP and FIB exhibited a positive correlation (Spearman correlation analysis, *R* = 0.73, *p* = 6.1 × 10^−14^) (Figure 1C). These results indicated a probable link between inflammation and coagulation. Moreover, the plasma concentrations of CRP and FIB were positively correlated with not only the viable CTC count but also with the percentage of circulating neutrophils (Figure 1D). Thus, neutrophils may play a crucial role in these intricate relationships. The relationships between viable CTC counts and other clinical characteristics, such as the pathological subtype, stage, presence of microscopic tumor embolus, and other hematological parameters, are shown in Figure S2.

Collectively, these results lead to the hypothesis that inflammation and hypercoagulability are favorable for CTC survival and that neutrophils act as a critical node linking inflammation and coagulation.

### Neutrophil Activation Was Related to CTC Survival

**Figure 2:**
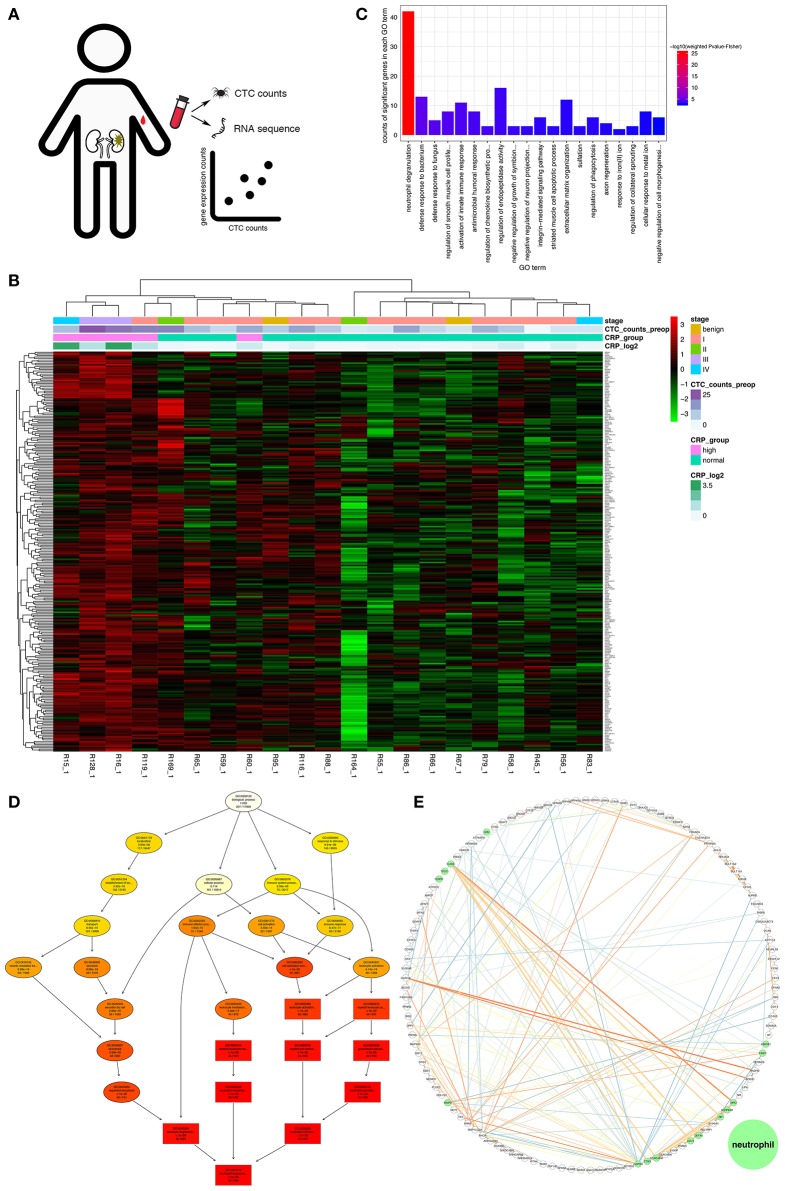
Gene profiles positively correlated with the CTC count. **(A)** Schematic of the discovery pipeline for genes of interest. **(B)** Heat map of the genes of interest. The heat map displays the log2 transformed TPM values of the 257 genes of interest. The x-axis shows the samples ordered by hierarchical clustering with Ward's linkage. The top bar on the x-axis represents the clinical stage of the patients, the second bar indicates the measured viable CTC count in 4 mL of preoperative peripheral blood, the third bar indicates the group stratified by the CRP concentration (cutoff of 0.6 mg/dL), and the fourth bar indicates the log2 transformed CRP concentration. **(C)** The 20 most significant GO terms enriched in the genes of interest. The vertical axis shows the number of positively correlated significantly enriched genes for each term. The horizontal axis displays the names of the GO terms ordered by the degree of significance. The color indicates the–log10 value (weighted Fisher's *p*-value). **(D)** Hierarchy chart of the 10 most significantly enriched GO biological process terms. The nodes are displayed in a circle or box shape and include information on the GO ID, GO name, weighted Fisher's *p*-values, and counts of genes of interest/total count of genes in the term. The box represents the 10 most significantly enriched terms. The colors of the box indicate the significance and range from dark red (most significant) to light yellow (least significant). The black arrows indicate is-a relationships. **(E)** The maximal subnetwork constructed with the genes of interest. The green nodes represent the nearest neighbors of ELANE. The thickness and color of the edges indicate the coexpression values; lower values have lower thicknesses and darker colors.

To investigate whether neutrophils acted as an assistant and linker in this process, transcriptomic analysis was performed on peripheral blood leukocytes from 21 treatment-naïve patients. At the molecular level, we explored the relationship between the leukocyte transcriptome and the viable CTC count and identified 257 genes positively correlated with the CTC count (see “Next-generation sequencing and data processing” within the Materials and Methods section for details; Figure 2A and Table S2). In addition, the 21 patients were divided into two groups by unsupervised clustering of the 257 genes (Figure 2B). Specifically, members of the group with higher expression levels of these 257 genes tended to have higher CTC counts and plasma CRP levels, but this group included patients in both early and late clinical stages of disease (Figure 2B). To annotate the functions enriched in the 257 genes of interest, GO analysis was performed. The 20 most significantly enriched biological process GO terms were associated with the innate immune response (particularly neutrophil degranulation and phagocytosis), extracellular matrix organization and the integrin-mediated signaling pathway (Figure 2C). Additionally, the GO hierarchy chart of the 10 most significantly enriched biological process terms converged into one term: neutrophil degranulation (GO: 0043312, Figure 2D). Furthermore, we extracted the maximal subnetwork constructed from the 257 genes of interest and highlighted the nearest neighbors of the ELANE gene, such as MPO and CTSG. The ELANE, MPO and CTSG genes encode elastase, MPO, and cathepsin G, respectively. These enzymes are not only released from primary azurophils during degranulation (39) but also decorate the meshwork of chromatin fibers in NETs (40) (Figure 2E). Thus, activated neutrophils are, to some extent, correlated with the survival of CTCs.

### NET Formation Cooperated With Coagulation to Promote CTC Survival

**Figure 3:**
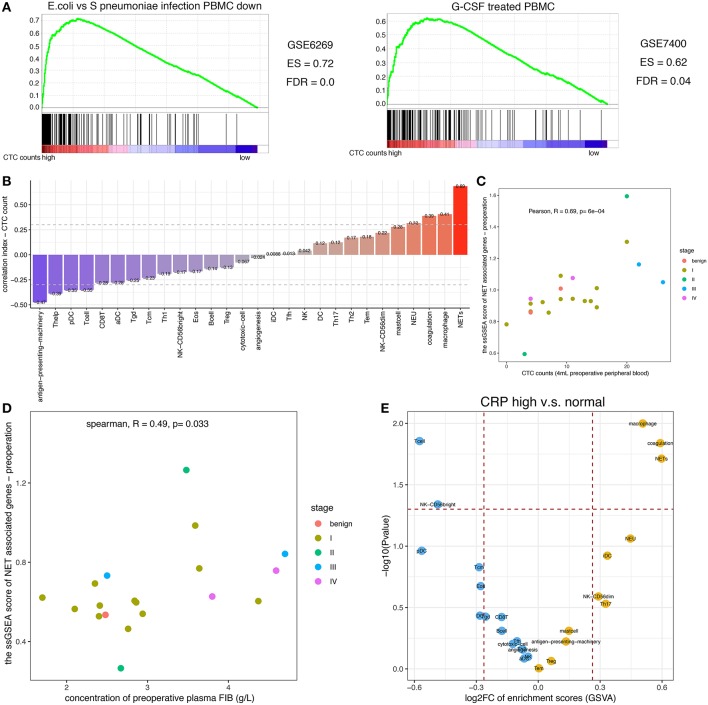
Relationship among the NET score, FIB level, and CTC count. **(A)** GSEA of the peripheral blood leukocyte RNAseq dataset. GSEA was performed with continuous-label CTC counts. The GSEA mountain plots exhibited the 2 most divergent signatures (with a minimum enrichment score of 0.6 and a false discovery rate (FDR) cutoff of 0.05). The gene set names were refined to fit the figure. **(B)** Relationship between the CTC count and the immunophenoscore as assessed by ssGSEA for immune cell subgroups and selected physiological processes. The vertical axis displays the correlation coefficients of the CTC counts and each subgroup of immune cells. In addition, the colors of the bars correspond to the correlation coefficient. **(C)** Relationship between the CTC count and the NET score. **(D)** Relationship between the NET score and the FIB concentration. **(E)** Plot of the enriched (yellow) and depleted (blue) immune phenotypes between the group with elevated CRP levels and the group with normal CRP levels based on the immunophenoscore (GSVA).

Because neutrophil-mediated inflammation was shown to promote CTC survival, we sought to determine whether NETs induced by circulating neutrophils were conducive to CTC metastasis. Indeed, the CTC count and expression value of each key molecule in NET production were positively correlated (Figure S3); particularly PAD4, an essential enzyme mediating histone hypercitrullination during NET formation (30). To further confirm this finding, we explored the data with another approach. When the CTC count was treated as a continuous variable, GSEA showed that the leukocytes in patients with high CTC counts were similar to *S. pneumoniae* rather than *E. coli* infection or to G-CSF-treated peripheral blood mononuclear cells (PBMCs) (Figure 3A). The *S. pneumoniae* infection signature suggested that low-density neutrophils (LDNs) copurified with mononuclear cells during density gradient centrifugation were activated (41). It has been acknowledged that unstimulated LDNs can produce NETs spontaneously (17, 42), and G-CSF can prime neutrophils to generate NETs (13). Additionally, we used gene set-based scoring systems (immunophenoscore obtained using the ssGSEA method; details are shown in the Materials and Methods section) to evaluate the functions and counts of physiological processes and immune cells. Interestingly, among the whole set of immune cells and their specific physiological processes, NET formation exhibited the strongest positive correlation with the viable CTC count (Pearson correlation analysis, *R* = 0.69, *p* = 6 × 10^−4^, Figures 3B,C). Increased neutrophil activity, particularly the NET formation mechanism, was unanimously associated with an increased viable CTC count.

**Figure 4:**
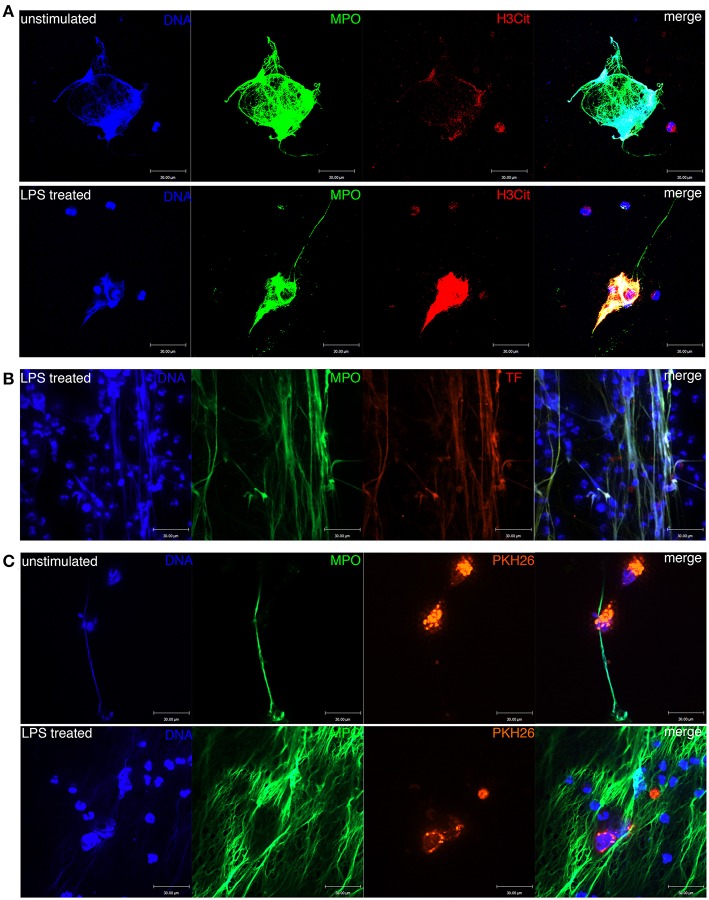
Formation of NETs in the peripheral blood of patients with ccRCC. **(A)** DNA, MPO, and H3Cit were colocalized with NETs formed in unstimulated and LPS-treated blood. **(B)** DNA, MPO, and TF were colocalized with NETs formed in LPS-treated blood. Neutrophil-derived TF was produced during NET formation. **(C)** NETs were entangled with ACHN cells in the blood of ccRCC patients.

Moreover, the NET score was positively correlated with the plasma concentration of FIB (Pearson correlation analysis, *R* = 0.49, *p* = 0.033, Figure 3D). Not only the transcriptomic data but also the image of immunofluorescence staining showed the similar result (Figure 4B) that NET-derived TF adheres to the DNA framework of NETs. Because TF is a factor that triggers an extrinsic coagulation cascade, eventually leading to crosslinking with fibrin, the defined list of genes involved in the coagulation system was composed of the key coagulation factors in the extrinsic coagulation pathway (details in the Materials and Methods section). The volcano plot of the immunophenoscores (GSVA scores) showed that the coagulation system and NET formation were stimulated consistently in patients with elevated CRP levels (Figure 3E). Thus, NETs may be the link between inflammation and coagulation.

### NETs Were Entangled With and Sheltered Spiked CTCs in the Circulatory System of RCC Patients

Next, we confirmed that NETs were morphologically detectable in unstimulated and LPS-treated peripheral blood leukocytes of RCC patients (Figure 4A). NETs were colocalized with released MPO and citrullinated histone 3 (H3Cit), which adhered to the extruded DNA scaffold. We also confirmed via immunofluorescence staining that the framework of NETs was decorated with NET-derived TF (Figure 4B). Thus, NETs function as a bridge to link inflammation and coagulation. Moreover, we observed NETs entangled with the spiked ACHN cells, which were labeled with PKH26, in both untreated and LPS-stimulated peripheral blood leukocytes of ccRCC patients (Figure 4C). Collectively, these results indicate that NETs induced by circulating neutrophils could form a protective shelter and vehicle conducive to the metastasis of CTCs.

### Inflammation and Coagulation Indexes in the Tumor Microenvironment Are Independent Prognostic Factors in RCC Patients

**Figure 5:**
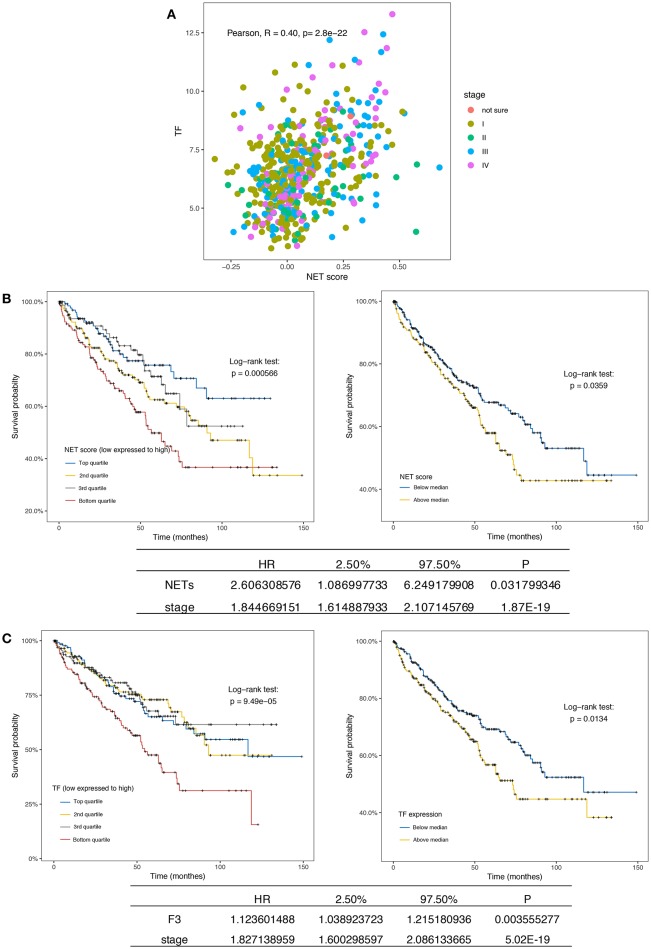
NETs and F3 in tissues are independent prognostic factors in ccRCC patients. **(A)** Kaplan-Meier plots of the overall survival of ccRCC patients (*n* = 533) from the TCGA database stratified by the NET score quartile (left, *p* = 0.000566) and median NET score (right, *p* = 0.0359). The bottom list shows the adjusted HR for NETs by clinical stage (HR = 16.18). **(B)** Kaplan-Meier plots of the overall survival of ccRCC patients (*n* = 533) from the TCGA database stratified by the TF expression quartile (left, *p* = 0.49 × 10^−5^) and median TF expression value (right, *p* = 0.0134). The bottom list shows the adjusted HR for TF expression by clinical stage (HR = 1.12). **(C)** The NET score and TF expression value were positively correlated.

Although persistent inflammation and hypercoagulability increased the counts of viable metastatic seeds (CTCs) in the circulatory system, we sought to determine whether these factors contribute to the generation of CTCs or the microenvironment of primary tumors. To this end, we investigated RNAseq data in the TCGA database from 533 patients with ccRCC. A marked separation of the survival curves was confirmed regardless of whether the samples were stratified by quartiles or median NET scores (*p* = 0.00056 and 0.0359, respectively). Furthermore, the NET score was an independent prognostic factor when the model was adjusted for clinical stage (Figure 5A). Interestingly, the triggering factor of the extrinsic coagulation pathway, TF, could separate the survival curves and was an independent prognostic factor (Figure 5B). In addition, the NET scores and TF expression values were positively correlated (Figure 5C). Via immunofluorescence, neutrophils were also observed to release NETs decorated with F3 (Figure 4B). In a sense, both NET-derived TF-mediated fibrin crosslinking and the DNA scaffold released from NETs create a secure scaffold for cancer cell invasion.

## Discussion

Most RCC patients die from cancer that relapses after CTCs spread to other organs rather than from early localized carcinoma. However, to date, little is known about the mechanisms by which CTCs survive in the circulatory system to seed other inhospitable tissues. Based on our research results, we propose a potential process by which circulating renal cancer cells metastasize to other organs from the perspective of dysregulated inflammation and coagulation.

NETs are the bridge connecting inflammation and coagulation, and the NET DNA scaffold not only provides a location for platelet and red blood cell adhesion (43) but is also decorated with TF (11). In a functioning host, the immune system cooperates with the coagulation system for infection defense or trauma repair to maintain homeostasis. Among the diverse defense mechanisms, immunothrombosis generated by immune cells and specific thrombosis-related molecules generate an intravascular scaffold that helps to defend against exogenous pathogens (44). However, cancer is known as the wound that does not heal (45). Thus, uncontrolled immunothrombosis in a cancer-burdened host might become a major pathological process fostering foreign CTCs.

Our data indicated that peripheral blood leukocytes of RCC patients tend to be activated by a specific process, similar to the spontaneous activation of LDNs or G-CSF priming *in vitro*—namely, the formation of NETs—particularly in samples with a higher viable CTC count. In addition, our results indicated that increased NET formation was accompanied by increased plasma levels of FIB, which have been reportedly associated with poor prognosis due to NK cell inhibition (46, 47), and several studies have reported that NET formation promotes fibrin and thrombin generation (48–51). Thus, intravascular neutrophils form TF-decorated tethers and slings to activate the coagulation system, simultaneously anchoring CTCs to these NETs as a protective vehicle.

Moreover, intravascular NETs not only protect CTCs from attack but also promote their extravasation, as dysregulated NET formation contributes to inflammatory vascular injury, endothelial cell shrinkage and tissue damage (50, 52), and NET-remodeled laminin activates integrin α3β1 signaling (18), which was a significantly enriched GO term associated with the 257 genes of interest herein (Figure 2C). NETs and their decorated molecules (e.g., TF), which were herein demonstrated to be indicators of poor prognosis in the TCGA ccRCC RNAseq data (Figure 5), may cooperate to facilitate cancer cell penetration into the vasculature. In addition, NETs have been shown to promote angiogenesis *in vitro* (49), but this phenomenon has not yet been observed *in vivo*.

Additionally, patients with microscopically visible microtumor thrombosis exhibited higher numbers of viable single CTCs than patients without visible thrombosis (Figure S2C). This finding, combined with the abovementioned information, revealed that large renal venous tumor thromboses originate from a single CTC captured by NETs and accumulate gradually. However, the hypothesis of venous tumor-immunothrombosis formation needs further validation. Except for venous tumor-immunothrombosis, research has suggested that the appearance of CTCs is a risk factor for venous thromboembolism (VTE) in early and metastatic breast cancer (53, 54). Similar to our result, viable CTC counts and plasma FIB displayed a positive correlation (Figure 1B).

RCC is not only complicated by thrombus but is also strongly associated with inflammation, and elevated levels of plasma CRP, an indicator of inflammation, predict poor survival in patients with localized RCC (55) and metastatic RCC (56). However, the mechanisms driving the association of CRP, an acute phase reactant protein produced by the liver, with cancer-specific survival are poorly understood. CRP levels have been reportedly correlated with the production of interleukin-6 (IL-6) (57), which mediates ongoing inflammatory processes. We showed that both persistent inflammation and the coagulation system were activated in patients with elevated CRP levels (Figure 3E). Thus, the presence of CRP, a non-specific indicator of inflammation, may represent a microenvironment suitable for NET formation and coagulation system activation, which is conducive to the intravascular survival of CTCs, metastatic seeds. Conversely, fewer alive CTCs were detected when peripheral blood dendritic cells (DCs) and antigen-presenting machinery were activated (Figure 3B). Consistent with conventional cognition, both the activated protumor immune response and the compromised function of the antitumor immune response (such as DCs) support the survival of CTCs (58).

In this study, we did not identify a direct relationship between the CTC count and prognosis due to the lack of complete follow-up information for the enrolled patients. We observed only spiked cancer cells entangled in NET tethers in untreated ccRCC patients (Figure 4B). We will attempt to elucidate the process by which immunothrombosis (NET entanglement with the fibrin network) is connected to CTCs *in vivo* in the future.

In conclusion, we proposed a survival mechanism of intravascular CTCs—intravascular neutrophils form TF-decorated tethers and slings, namely, NETs, to activate the coagulation system and further anchor CTCs. Thus, degrading this framework (the double shelter of CTCs—the DNA scaffold and the fibrin network initiated by NET-derived TF) may be a potential way to inhibit metastasis.

## Data Availability

The data for the 21 raw RNA sequences generated for this study can be found in the Genome Sequence Archive (59) at the BIG Data Center (60), Beijing Institute of Genomics (BIG), Chines Academy of Sciences, under accession number HRA000042. [http://bigd.big.ac.cn/gsa-human]. Data download needs to be in agreement with the corresponding author.

## Ethics Statement

This study was carried out in accordance with the recommendations of the Ethics Committee of the Cancer Institute and Hospital of the Chinese Academy of Medical Sciences with written informed consent from all subjects. All subjects gave written informed consent in accordance with the Declaration of Helsinki. The protocol was approved by the Ethics Committee of the Cancer Institute and Hospital of the Chinese Academy of Medical Sciences.

## Author Contributions

JS, LF, and KZ contributed to the conception and design of the study. LW, YL and WJ collected the samples and clinical information. LG performed the experiments. XD provided instruction for the experiments. LF and LG performed the statistical analysis. LG wrote the first draft of the manuscript. LF, WZ, JM, KZ, and JS wrote sections of the manuscript. All authors contributed to the manuscript revision and read and approved the submitted version.

### Conflict of Interest Statement

The authors declare that the research was conducted in the absence of any commercial or financial relationships that could be construed as a potential conflict of interest.
