# Supplementary material for: Cooperation Between the Inflammation and Coagulation Systems Promotes the Survival of Circulating Tumor Cells in Renal Cell Carcinoma Patients
Source: Front Oncol. 2019 Jun 17;9:504. doi: 10.3389/fonc.2019.00504 (PMC6590108; doi:10.3389/fonc.2019.00504)
Supplement: Supplementary file 1 [file Data_Sheet_1.docx]

Supplementary Material

#
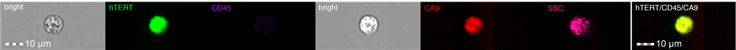
Supplementary Figure

**Figure S1.** **Image of CTCs in a treatment-naïve renal carcinoma patient.** SSC: side scatter.

**
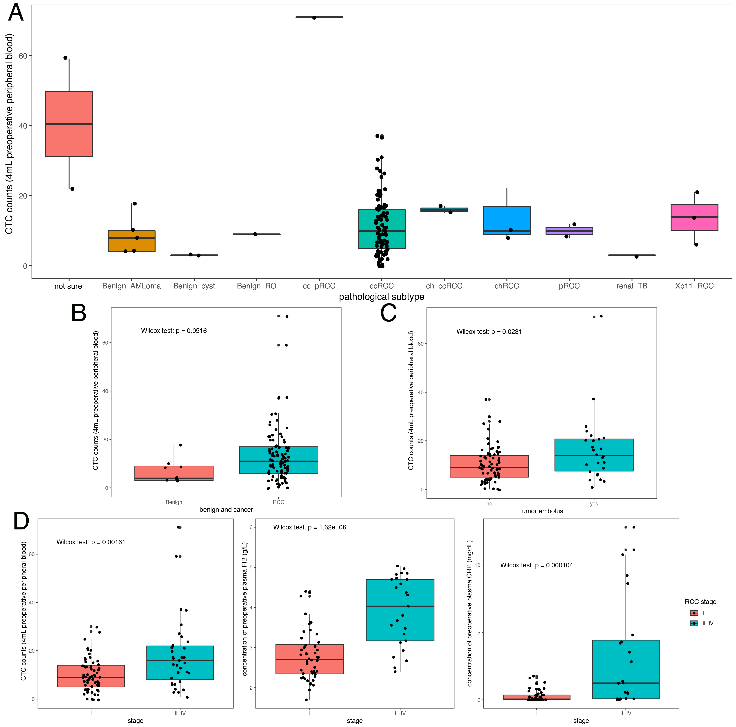
**

**Figure S2 Relationship between the viable CTC count and other clinical indexes.** (A) Distribution of CTC counts in various pathological subtypes. Annotations on the x-axis: unoperated patients with an undetermined pathological subtype (not sure); renal angiomyolipoma (Benign_AMLoma); renal cyst (Benign_cyst); renal oncocytoma (Benign_RO); clear cell renal cell carcinoma (ccRCC); chromophobe renal cell carcinoma (chRCC); papillary renal cell carcinoma (pRCC); Xp11 transposition-associated renal cell carcinoma (Xp11_RCC); coexisting ccRCC and pRCC (cc_pRCC); coexisting ch_RCC and ccRCC (ch_ccRCC); nephrotuberculosis (renal_TB). (B) CTC counts were higher in patients with benign than in patients with carcinoma. (C) CTC counts were higher in with patients with microscopically observable tumor embolus than in those without. (D) Differences in CTC counts and FIB and CRP levels in stage I to stage II-IV RCC.

**
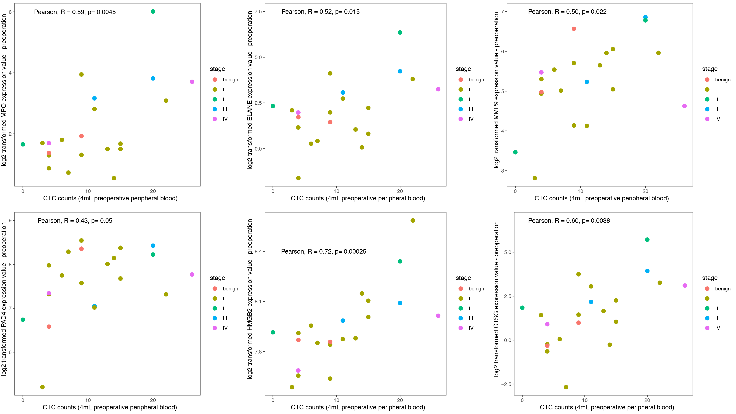
**

**Figure S3 Relationship between the CTC count and each key molecule in NET formation.**
